# Launching the Clostridioides difficile collection

**DOI:** 10.1099/jmm.0.001877

**Published:** 2024-08-20

**Authors:** Lovleen Tina Joshi, Stephen Michell, Ed Kuijper

**Affiliations:** 1 University of Plymouth, Plymouth, UK; 2 University of Exeter, Exeter, UK; 3 Leiden University Medical Center, Leiden, Netherlands

**Keywords:** *Clostridioides difficile*, collection, One Health, spores, transmission, updates

*Clostridioides difficile* is an anaerobic, Gram-positive spore-forming bacterium that is the primary cause of antibiotic-associated diarrhoea in humans, both in healthcare settings and in the community [1,2]. *C. difficile* infection (CDI) is usually caused when broad-spectrum antibiotic exposure disrupts the colonic microbiota, allowing *C. difficile* to colonize and infect patients. The role of the gut microbiome is extremely important and offers new therapeutic interventions to treat and prevent CDI. CDI often results in long hospital stays and poor patient outcomes in susceptible patients [1,2]. CDI causes ~29 000 deaths per year in the USA and 8382 deaths per year in Europe, and current data suggest that the burden of CDI is high [3]. The use of broad-spectrum molecular diagnostic tests to detect microbiological pathogens causing diarrhoea has contributed to a better insight into the role of *C. difficile* in human diseases, especially among community patients without clear risk factors for CDI [4].

Despite the burden and high incidence of CDI, there has been little international focus on the importance of this Gram-positive micro-organism in infectious diseases and antimicrobial resistance. Microbial evolution occurs in response to natural and artificial stressors, and *C. difficile* is no exception. The organism is fascinating in its ability to survive harsh environments, produce spores and resist the effects of antibiotic treatment. In addition, the recent discovery of metronidazole resistance, vancomycin resistance and fidaxomicin resistance in *C. difficile* is a cause for concern for effective treatment of CDI and highlights the need to understand the role of antibiotic resistance and its effects on CDI and recurrent CDI [5,8]. Spores are implicated in the spread of *C. difficile*, and effective infection control procedures are necessary to break the chain of *C. difficile* transmission. CDI is considered a One Health problem with the involvement of specific types of *C. difficile* that bridge the human–animal species border [9].

The role of *C. difficile* in infection is complex, and there is a need to review current knowledge and research methods, including the development of specific vaccines, new therapeutic and prophylactic approaches, patient education and understanding of the spread and epidemiology of *C. difficile. C. difficile* is continuously evolving with the recognition and emergence of new so-called hypervirulent types, such as the recently found PCR ribotype 955 in the UK [10].

Therefore, the Microbiology Society’s *Journal of Medical Microbiology* is pleased to announce the launch of a new *C. difficile* collection, which brings together and showcases the latest international research developments in the area. Topics within this collection range from *C. difficile* interaction with host microbiota to One Health, CDI animal and human challenge models, disinfection, testing and detection from leading *C. difficile* researchers, forming a comprehensive and diverse collection.

We are proud to curate this collection, and it gives us great pleasure to highlight the *C. difficile* pathogen within the *Journal of Medical Microbiology*. We welcome further contributions from scientists and clinicians with an interest in *C. difficile*, particularly in the remit of CDI, and we look forward to receiving your manuscripts. We wish to thank all authors and contributors who have dedicated their time to support this collection and hope that readers will enjoy the content.

## Abbreviations

CDI: *C. difficile* infection
PCR: polymerase chain reaction
